# Charing Cross Hospital

**Published:** 1916-05-20

**Authors:** 

**Affiliations:** Treasurer. George Verity, Chairman.


**Charing Cross Hospital.**

*To the Editor of* The Hospital.

Sir,—As it is obviously the duty—the necessary duty of all those in charge of hospitals such as the Charing Cross—to give their primary consideration to economy with a view of utilising all moneys so generously subscribed to the hospitals, it is considered by the Council of the Charing Cross Hospital that the large amount involved in printing the annual report (amounting to somewhere between £250 and £300) should be abandoned. A certain number will be printed, and upon the application of any subscriber who wishes to have one it will be posted to him, or should he at any time wish to see the report it will always be open to his inspection at the hospital.

Hoping that all subscribers will agree with this suggestion, we have the honour to be, yours obediently,

Lonsdale, Treasurer.

May 1916. George Verity, Chairman.

